# Mendelian randomisation highlights type 1 diabetes as a causal determinant of idiopathic pulmonary fibrosis

**DOI:** 10.1186/s13098-024-01331-x

**Published:** 2024-04-24

**Authors:** Xinlai Ma, Yang Zhang, Fan Wu, Xue Liu, Wei Zhang

**Affiliations:** 1 The First Clinical Medical College, Shandong University of Traditional Chinese Medicine, Jinan, China; 2 Department of Pulmonary and Critical Care Medicine, Affiliated Hospital of Shandong University of Traditional Chinese Medicine, No.42, West Culture Road, Lixia District, 250011 Jinan, Shandong China

**Keywords:** Type 1 diabetes, Idiopathic pulmonary fibrosis, Mendelian randomization

## Abstract

**Background:**

It is unclear whether type 1 diabetes (T1D) causes idiopathic pulmonary fibrosis (IPF), despite observational research linking the two conditions. Therefore, our study aimed to examine the causal link between T1D and the likelihood of IPF by employing the Mendelian randomization (MR) technique of two-sample Mendelian randomization.

**Methods:**

Using data from two genome-wide association studies (GWAS) with European ancestry, we performed a two-sample MR analysis. These studies involved 18,856 individuals (6,683 cases and 12,173 controls) for T1D and 198,014 individuals (10,028 cases and 196,986 controls) for IPF. We utilized inverse-variance weighted (IVW) analysis as our main approach to determine the association between the risk of IPF and T1D. To evaluate multidirectionality, the MR-Egger regression test was utilized, whereas heterogeneity was assessed using Cochran’s Q test. Additionally, a leave-one-out analysis was performed to assess the reliability of the results.

**Results:**

38 SNPs linked to T1D were employed as instrumental variables (IVs). Multiple MR methods yielded consistent results, and the MR analysis reveals a significant and positive causal impact of T1D on IPF (MR-IVW, odds ratio [OR] = 1.128, 95% confidence interval [CI] 1.034–1.230; P *=* 0.006). The limitations of the study include the lack of data from non-European groups and the inability to rule out the possibility of small links. Larger MR experiments are necessary to investigate minute impacts.

**Conclusions:**

The results of this study provide evidence that T1D contributes to the onset and advancement of IPF. This finding may provide important insights into the cause of IPF and possible treatments in the future.

**Supplementary Information:**

The online version contains supplementary material available at 10.1186/s13098-024-01331-x.

## Introduction

IPF is an incurable and worsening respiratory condition of unknown cause, characterized by the progressive deterioration of lung function. The median life expectancy for individuals with IPF is a mere 3.8 years [1, 2]. The progression of disease involves damage to alveolar epithelial cells, a transition from epithelial to mesenchymal cells, and the activation of fibroblasts, resulting in an elevated accumulation of extracellular matrix proteins. Alveolar architecture is remarkably disrupted and the extracellular cell matrix is changed, replacing normal healthy lung tissues. These alterations eventually result in a significant disturbance of the carefully regulated gas exchange mechanism and a decrease in lung compliance, which finally induce respiratory failure and mortality [3]. There is no cure for IPF and treatment options are limited. Therefore, a comprehensive comprehension of coexisting conditions in the IPF population, specifically their correlation, has the potential to greatly enhance medical treatment, ultimately leading to better chances of survival and improved quality of life [4]. Common comorbidities in IPF include respiratory diseases (e.g., emphysema, COPD, obstructive sleep apnea, and lung cancer), cardiovascular diseases (e.g., ischemic heart disease, pulmonary hypertension, and coronary artery disease), gastrointestinal diseases (e.g., gastroesophageal reflux disease and esophageal hiatal hernia), and metabolic diseases (e.g., diabetes mellitus and hypothyroidism) [5]. The occurrence of T1D is significantly greater among individuals with IPF compared to the overall populace, and T1D is linked to an elevated susceptibility to IPF [6]. There are clinical studies showing significant clinical and histopathological correlations between IPF and T1D.The formation of fibrosis is thought to be associated with reactive oxygen species and advanced glycation end products that arise from high blood sugar levels [7]. T1D and IPF are characterised by disorders of the immune system that can trigger chronic inflammation in any organ system. Abnormal leukocyte telomere length has the potential to increase the risk of T1D and IPF [8]. Therefore, it can serve as a predictor and may provide new potential therapeutic targets for T1D and IPF. However, altered leukocyte telomere length may not be a direct cause of T1D and IPF [8]. Previous observational studies investigating the relationship between endocrine and metabolic factors and IPF have yielded inconsistent results. Therefore, there is a need for a more comprehensive study of the effects of endocrine metabolic factors on IPF using Mendelian randomization (MR) analysis [9].

Although randomized controlled trials are considered the benchmark for evaluating causality, their application is restricted due to practical constraints, expenses, and ethical factors [10]. IPF is relatively rare, making it unfeasible to collect a large sample in a longitudinal study to adequately examine the relationship between T1D and IPF. In addition, many known and unknown confounding factors pose a great challenge in studying the causal effect of T1D on IPF. The emergence of causal inferences between exposures and observational findings offers this possibility. The study of causality using genetic instruments (e.g., single nucleotide polymorphisms [SNPs]) can be effectively conducted with the help of MR framework [11]. It is similar to a “natural” randomized controlled trial, where the random assignment of genetic alleles affecting exposure largely eliminates the effects of unobserved confounders and avoids reverse causality and measurement error, which may affect other study designs [12]. The availability of publicly accessible GWAS data has resulted in the generation of comprehensive summary-level information. As a result, two-sample MR has emerged as a cost-effective and efficient approach to investigate causal connections between health risk factors and disease outcomes [13].

Using publicly accessible GWAS summary statistics, we investigated the causal association between genetically predicted T1D and IPF within an MR framework in this study. The results may offer additional evidence for the etiology of IPF.

## Method

### The methodology and the origin of the data

**Fig. 1:**
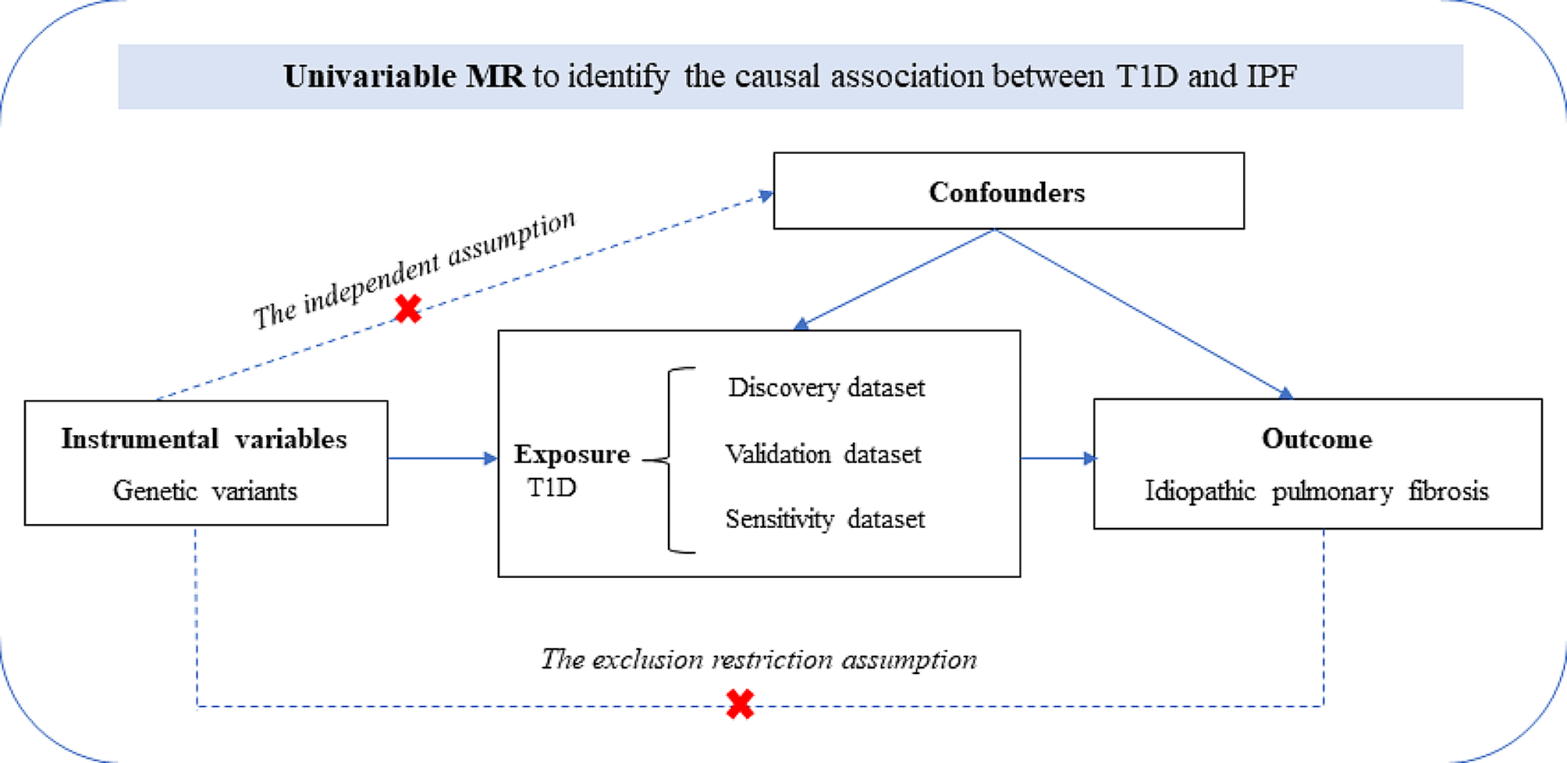
Study design of univariable MR to identify the causal association between T1D and IPF. IPF, idiopathic pulmonary fibrosis; MR, Mendelian randomisation

We performed a two-sample MR analysis using combined GWAS data to evaluate the possible causal association between T1D and IPF. All GWAS studies included unrelated individuals of European ancestry. A total of 18,856 participants (6,683 cases and 12,173 controls) from the European Bioinformatics Institute (EBI) were part of this study, and genome-wide genotypes were measured. The statistical data for IPF were acquired from the GWAS summarized by Dhindsa et al. [14] and colleagues. The GWAS analysis consisted of 1028 IPF patients and 196,986 controls. To estimate and test for causal effects on outcomes (i.e., IPF), the MR framework employed independent instrumental SNPs as instrumental variables (IVs) for exposures (e.g., T1D). To ensure the validity of instrumental variables, three modeling assumptions are necessary for standard MR analysis. (i) IV is associated with T1D at a genome-wide significant level; (ii) IV must be independent of any confounding factors; (iii.) IV affects IPF only through T1D (Fig. 1).

### Genetic instrumental variables

In order to satisfy the initial hypothesis of the MR analysis, which states that instrumental variables (IVs) have a strong connection with T1D biomarkers, we carefully chose independent IVs that exhibited statistically significant correlations with T1D at the genome-wide level (*P* < 5 × 10 − 8, linkage disequilibrium < 0.001, genetic distance = 10, 000 KB) [15]. To avoid potential confounding effects of genetic variation, we employed RStudio 4.3.2 and the TwoSampleMR package to detect 38 SNPs that are significantly linked to T1D (Additional file 1), meeting the initial hypothesis. To evaluate if the IVs included were linked to any recognized confounders, we conducted a search in the PhenoScanner database (http://www.phenoscanner.medschl.cam.ac.uk). Risk factors for IPF have been identified, such as smoking, dust exposure, and reflux esophagitis [1]. Hence, in this research, we eliminated the SNP associated with smoking (rs3184504), and subsequently collected information from IPF GWAS for 37 out of the 38 mentioned SNPs. Finally, we used 37 SNPs as instrumental variables for T1D in our study. Furthermore, we computed the F-value (F = beta^2/se^2) [16] to verify that the included independent variables are not affected by feeble IVs [17].

### Statistical analysis

Many robust statistical methods were utilized to ensure the accuracy and dependability of the findings, and sensitivity analyses were conducted to assess the potential impact of various sources of bias. To assess the impact of a 1 standard deviation (SD) rise in standardized logarithmically transformed T1D on IPF vulnerability, the primary analytical approach employed was the MR inverse variance weighting (MR-IVW) technique [18].We conducted four additional sensitivity analyses using the weighted-median approach [19], the MR-Egger method [20], the weighted mode [21], and the simple mode [22] to assess the strength of the results. Reliable estimates were obtained using the weighted median method when valid IV accounted for over 50% of the information. We evaluated various instrumental variables (IVs) to examine horizontal pleiotropy, employing the MR-Egger method [20]. Furthermore, we investigated the diversity among particular independent variables by utilizing Cochrane’s Q-value [23]. Additionally, we conducted a sensitivity analysis by leaving out one SNP at a time to investigate any potential unequal impacts of individual SNPs on the overall estimation. *P*-values ranging from the Bonferroni correction to 0.05 were deemed indicative of a potential association warranting additional scrutiny. We utilized the “MendelianRandomization” [24], “MRPRESSO“ [25], and “TwoSampleMR” R packages for the analysis, which was carried out in the R environment (version 4.3.2). A significance threshold of 0.05 was applied.

## Results

**Fig. 2:**
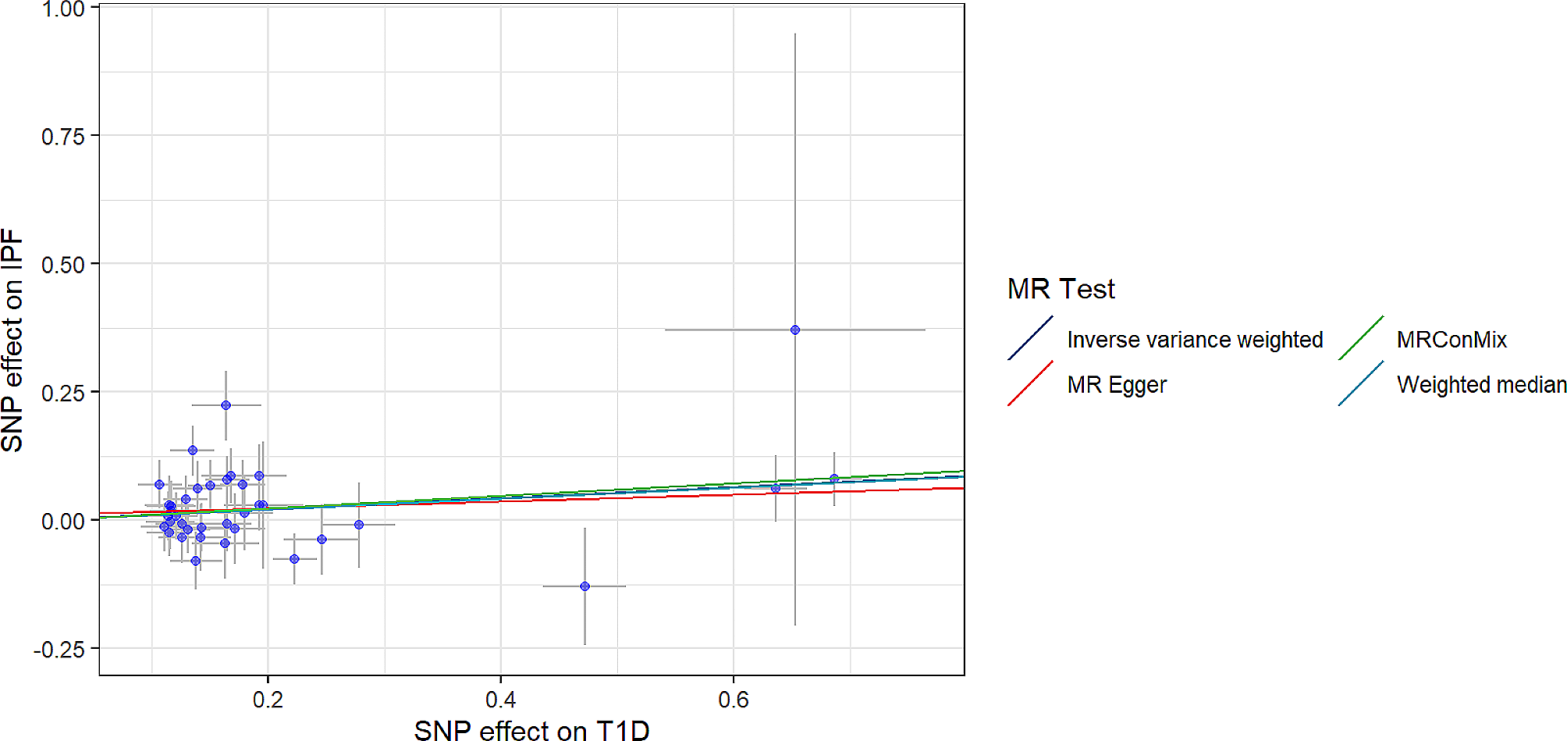
Scatter plot for the causal effect of T1D on IPF risk The extent of the cause-and-effect relationship is demonstrated by the incline of the linear graph…

**Fig. 3:**
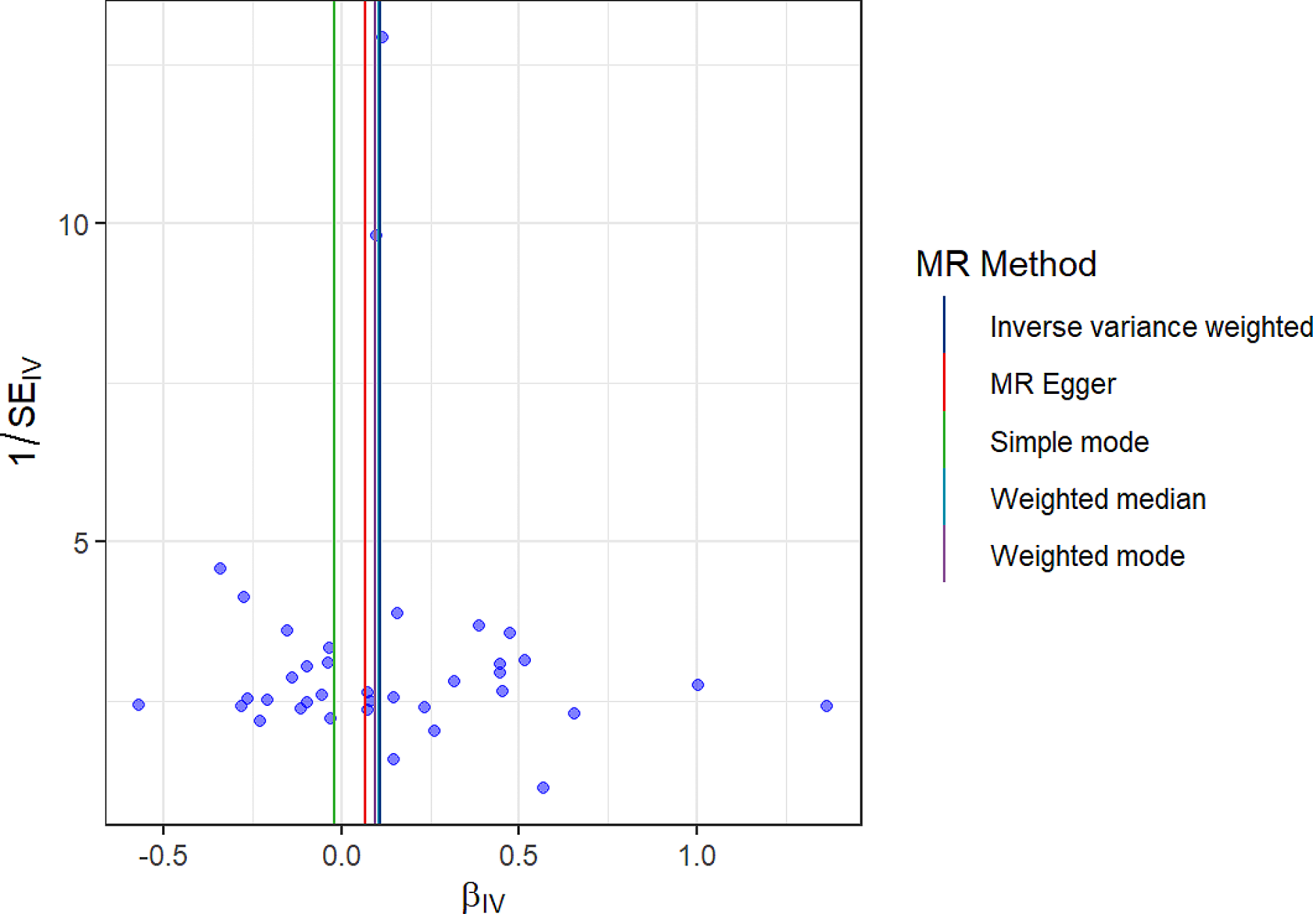
Funnel plot for the the general heterogeneity in the impact of T1D on the risk of IPF.

**Fig. 4:**
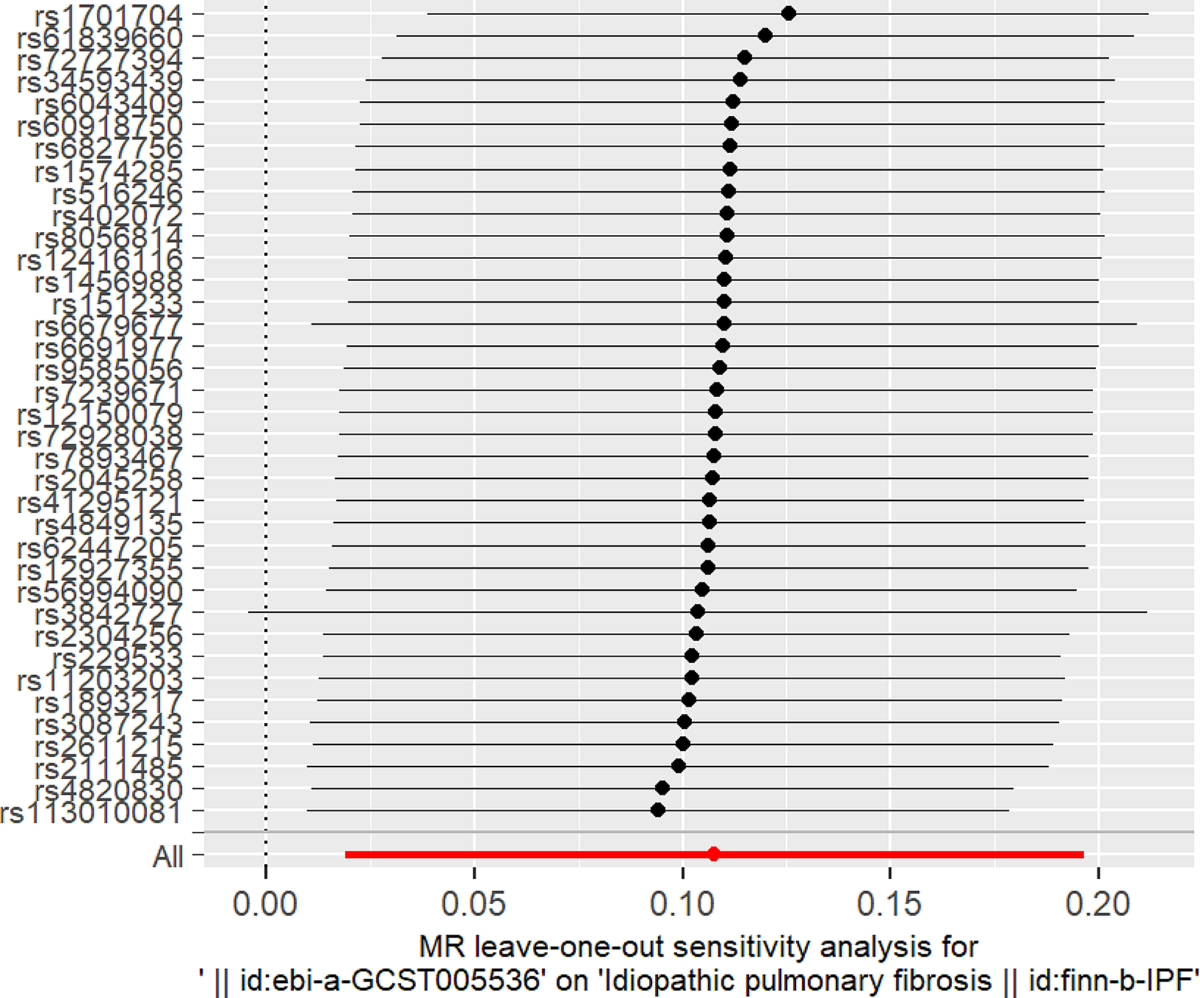
Forest plot for leave-one-out analysis, where each point on the left side represents the causal effect determined by IVW after removing the specific SNP. IVW, inverse variance weighted method

To assess the genetic relationship between T1D and IPF, we obtained 37 SNPs as IVs and used them in an MR analysis. The IVW method yielded significant evidence of a causal link between T1D and the likelihood of IPF, with an OR of 1.1137 (MR-IVW) and a 95% CI of 1.019089–1.217114 (*P* = 0.017434) (Table 1). This indicates that a self-reported history of T1D could lead to an average 11.37% increased risk of IPF, as depicted in the scatter plot (Fig. 2).The F-statistics > 10 for each SNP suggested a low level of instrumental variable bias. Furthermore, our results were validated through a sensitivity analysis, which revealed no variation among independent variables according to the Cochran’s Q test results (PIVW = 0.282977, PMR Egger = 0.259668, Table 2). The symmetrical nature of the funnel plot provided additional evidence for the absence of heterogeneity(Fig. 3).Moreover, the MR-Egger regression findings indicated the absence of significant pleiotropy for any instrumental variable (*P* = 0.54392, Table 2). This implies that the influence of instrumental variables on IPF through mechanisms unrelated to T1D is unlikely. Consistently similar results were obtained from the leave-one-out sensitivity analysis, even when removing one SNP at a time (Fig. 4). Overall, several MR techniques consistently estimated the causal link between T1D and IPF.

**Table 1:** MR analysis results between T1D and IPF.

Method	SNPs	b	SE	P	OR(95%CI)
IVW	37	0.107	0.045	0.017	1.113(1.019–1.217)
MR Egger	37	0.066	0.080	0.412	1.069(0.912–1.252)
Weighted median	37	0.106	0.064	0.100	1.112(0.979–1.263)

T1D: Type 1 diabetes; IPF: idiopathic pulmonary fibrosis; SNP: single-nucleotide polymorphism; OR: odds ratio; 95%CI, The 95% confidence intervals

**Table 2:** Heterogeneity and Pleiotropy tests of MR.

Test	Method	Effect size	P
Heterogeneity	Q MR Egger	39.946	0.259
	Q IVW	40.375	0.282
Pleiotropy	MR-Egger regression	0.0103	0.543

Q: Cochran’s Q test; MR: Mendelian randomization; IVW: inverse variance weighted

## Discussion

Using a two-sample MR design, we demonstrate the causal connection between T1D and IPF in this research. The results are robust and convincing, supported by the confirmation of the findings through different MR approaches with diverse modeling assumptions and an independent validation set. Furthermore, no indications of reverse causation were found in the two-way investigation. This study benefited from the extensive GWAS sample size, including 1028 cases and 196,986 controls, and fully utilized MR analysis with genetic instruments. Our research confirms the results of previous observational studies that associated T1D with pulmonary fibrosis [6].

Mounting evidence suggests that diabetes can exert a substantial influence on pulmonary health. The cause of lung damage in diabetic individuals is still a topic of debate. It is suggested that the development of T1D may involve complex underlying processes IPF. Studies have shown that a significant increase in blood sugar can lead to a gradual decline in the function of pancreatic β-cells and an increase in insulin resistance. This process is referred to as “glucose toxicity“ [26]. This can trigger oxidative stress, activate the JNK pathway, and potentially lead to lung fibroblast activation, further promoting pulmonary fibrosis [27]. Another study suggests that diabetes-related interactions between platelets and endothelial cells may contribute to oxidative damage and persistent vascular inflammation, leading to pulmonary fibrosis [28]. TGF-β’s pro-fibrotic characteristics are mediated by connective tissue growth factor (CTGF), which is crucial for fibrosis and tissue remodeling. The kidney, heart, liver, skin, and lungs are among the fibrotic organs where CTGF expression has been shown to be upregulated [29]. A study on the detrimental consequences of diabetes on pulmonary fibrosis used a streptozotocin-induced diabetic rat (STZ rat) model, in which the lung tissues had a considerably higher expression level of CTGF. Furthermore, the levels of CTGF and transcription activator in the lung tissues of diabetic rats were reversed through the treatment of hyperglycemia [30]. A study by Fariña et al. [31] suggests that diabetes can disrupt the alveolar-capillary barrier’s basement membrane, leading to excessive collagen and extracellular matrix deposition, as well as inflammatory cell infiltration, all of which can exacerbate fibrotic changes.

There are multiple advantages to our research. This is the initial study to investigate the possible connection between T1D and the likelihood of IPF, establishing a cause-and-effect association between the two ailments. Additionally, the GWAS data from two large European population samples formed the basis of this MR analysis, providing sufficient power to establish causality. Furthermore, T1D has a lasting influence on the risk of IPF, as demonstrated through MR analysis, and this impact is improbable to be affected by any confounding variables.

Nevertheless, the study has its constraints. Our findings may not be applicable to populations of diverse ethnic backgrounds, as they primarily rely on participants of European descent. Additionally, while horizontal pleiotropy was not detected, the precise purpose of most of these SNPs remains unclear. This reduces statistical efficiency by increasing the residuals, even though it may not significantly affect overall causality. Ultimately, the frequency of IPF rises considerably with advancing age [32], and relying on combined information from GWAS rather than individual-level data complicates the establishment of a direct cause-and-effect connection between T1D and age-specific IPF. Furthermore, as this information was not relevant to each person in all datasets, the IPF GWAS in this research could not be modified for age and gender. In conclusion, the MR findings suggest that genetic predisposition to T1D has an influence on the risk of IPF throughout one’s lifetime. Consequently, additional investigation is required to ascertain the immediate consequences of T1D on the risk of IPF.

## Conclusions

In conclusion, our study provides compelling evidence that the self-reported history of T1D is a causative factor that influences the risk of IPF. This discovery may offer new insights into the etiology of IPF. Importantly, T1D is a medically significant mechanism that requires careful monitoring in IPF patients. However, treating IPF based on these findings may not be easy, and further pathological and biochemical research is definitely needed to fully comprehend the complex connection between T1D and an increased risk of IPF.

### Electronic supplementary material

Below is the link to the electronic supplementary material.

Supplementary Material 1

## Data Availability

No datasets were generated or analysed during the current study.
